# Supervised Object-Specific Distance Estimation from Monocular Images for Autonomous Driving

**DOI:** 10.3390/s22228846

**Published:** 2022-11-16

**Authors:** Yury Davydov, Wen-Hui Chen, Yu-Chen Lin

**Affiliations:** 1 Graduate Institute of Automation Technology, National Taipei University of Technology, Taipei 10608, Taiwan; 2 Department of Automatic Control Engineering, Feng Chia University, Taichung 40724, Taiwan

**Keywords:** monocular depth estimation, autonomous driving, computer vision, convolutional neural networks

## Abstract

Accurate distance estimation is a requirement for advanced driver assistance systems (ADAS) to provide drivers with safety-related functions such as adaptive cruise control and collision avoidance. Radars and lidars can be used for providing distance information; however, they are either expensive or provide poor object information compared to image sensors. In this study, we propose a lightweight convolutional deep learning model that can extract object-specific distance information from monocular images. We explore a variety of training and five structural settings of the model and conduct various tests on the KITTI dataset for evaluating seven different road agents, namely, person, bicycle, car, motorcycle, bus, train, and truck. Additionally, in all experiments, a comparison with the Monodepth2 model is carried out. Experimental results show that the proposed model outperforms Monodepth2 by 15% in terms of the average weighted mean absolute error (MAE).

## 1. Introduction

Distance estimation is a highly relevant task in the context of autonomous driving. A set of requirements must be met in order to consider a distance estimation system suitable for driving applications, namely, a low error magnitude and a high response rate. Unless these conditions are met, the developed distance estimation system cannot ensure the safety of passengers. Deep neural networks capable of extracting depth or distance information from monocular images are a promising approach to the described problem due to their ease of deployment and relatively low hardware requirements. To explore the existing approaches to monocular distance estimation and their limitations, we provide a brief overview of different deep learning models from the literature.

The most vivid branch of monocular depth estimation algorithms consists of supervised, semi-supervised or self-supervised models that construct depth or disparity maps from input images, i.e., by assigning a relative or absolute distance value to each input pixel. Several of the most well-known examples of this approach are presented in ref. [1,2]. The Monodepth and Monodepth2 models utilize a combination of a residual feature extractor and a pose estimation network that extracts pose deviations between pairs of input images. The features produced by these networks are processed to extract disparity information in a self-supervised fashion using pixel-wise minimum reprojection loss. The constructed disparity maps can be further converted into depth maps using the information about the hardware settings used during data collection. These models are trained and evaluated on the KITTI [3] dataset. Two other notable self-supervised depth estimation models are described in ref. [4,5]. The model by Guzilini et al. utilizes 3D convolutions to extract depth information from 3D geometric constraints, and the model proposed by Zhao et al. leverages Siamese networks to achieve the same goal. Self-supervised methods in general are highly valuable, as they facilitate the use of large unlabeled datasets, eliminating the need for complicated ground truth depth collection. However, they usually suffer from generalization issues [6], meaning that they perform significantly worse on data obtained in scenarios that differ from those present in the training data. An attempt to overcome this limitation can be found in the recent MiDaS model [7]; while conceptually similar to the ones mentioned above, MiDaS is trained on multiple datasets. This significantly improves the model’s generalization capabilities. However, this advantage comes at a cost of losing the ability to generate metric depth maps; the MiDaS model is capable of generating disparity maps only.

Several supervised methods of estimating pixel-wise depth should be mentioned here as well. For example, Lee et al. [8] proposed a supervised depth estimation model built around Local Planar Guidance (LPG) layers and Dense and Contextual Feature Extractors (DFE, CFE). Another supervised model of this kind can be found in ref. [9], where the authors employed a Spacing-Increasing Discretization approach to generate high resolution depth maps. In ref. [10], the authors developed the Attention-Based Context Aggregation Network, which gives their model an advantage in building pixel-wise contextual relationships, increasing the depth prediction accuracy. While more effective in terms of generalization performance, supervised depth estimation algorithms require the collection of ground truth depth information, which is a hard and expensive task. In addition, they depend on the availability of specialized devices (i.e., LIDAR sensors).

To address the ground truth collection complications, a large and diverse set of models that do not work with depth maps have been developed in the field of monocular depth estimation. To distinguish these approaches, we refer to them as distance estimation algorithms. Such approaches include, for example, models that work with specific camera types, i.e., fisheye cameras (see [11]), or utilize camera motion to extract distance information [12]. The latter, however, suffers significantly from the irregular motion problem; in a real-world driving setting, the car is moving unevenly, especially in dense traffic, which may severely affect the accuracy of distance prediction. Finally, several authors have proposed a somewhat simpler yet potentially effective approach based on using object-specific distance information. Such models, examples of which can be found in ref. [13,14], combine an object detection model with a distance estimation module that learns to predict distances from the detected objects’ dimensions and types.

In this paper, we propose an approach that can be attributed to the object-specific distance estimation algorithm group. Our main contributions include:

− A convolutional neural network model capable of extracting distance information from the dimensions (represented by bounding boxes) and the appearance of road agents while retaining a relatively low number of parameters and a high inference speed;
− A data-preprocessing technique that forces the network to learn object-specific features and that further increases its response rate;
− An optics-based method of converting the proposed network’s outputs to metric distance values that takes into account bounding box uncertainties;
− A comprehensive study of the data augmentation and sampling techniques as well as structural settings that can improve the model’s performance on a heavily imbalanced dataset.

The bounding boxes used as inputs for the model are obtained with the YOLO-v5 object detector. The model is trained and evaluated on the KITTI benchmark, for which the details of data processing and analysis are presented in Section 2. After considering multiple design features of the model in Section 2, we further test different configurations of the model’s hyperparameters in Section 3. Every configuration is additionally tested against the Monodepth2 model. The results are discussed in Section 4 and summarized in Section 5.

## 2. Materials and Methods

### 2.1. Preprocessing and Analysis of the KITTI Dataset

As stated above, the KITTI dataset was chosen for the proposed model’s training and evaluation. This choice was largely motivated by the popularity of this benchmark among the research groups working on monocular depth estimation, which stems from its large size and high-quality ground truth depth information. As we compare the developed model to the Monodepth2 model in terms of performance, we use the Eigensplit [15] of the dataset. This comes at the disadvantage of being unable to balance the testing data, which is addressed by using a weighted performance metric that we introduce in further detail.

**Figure 1:**
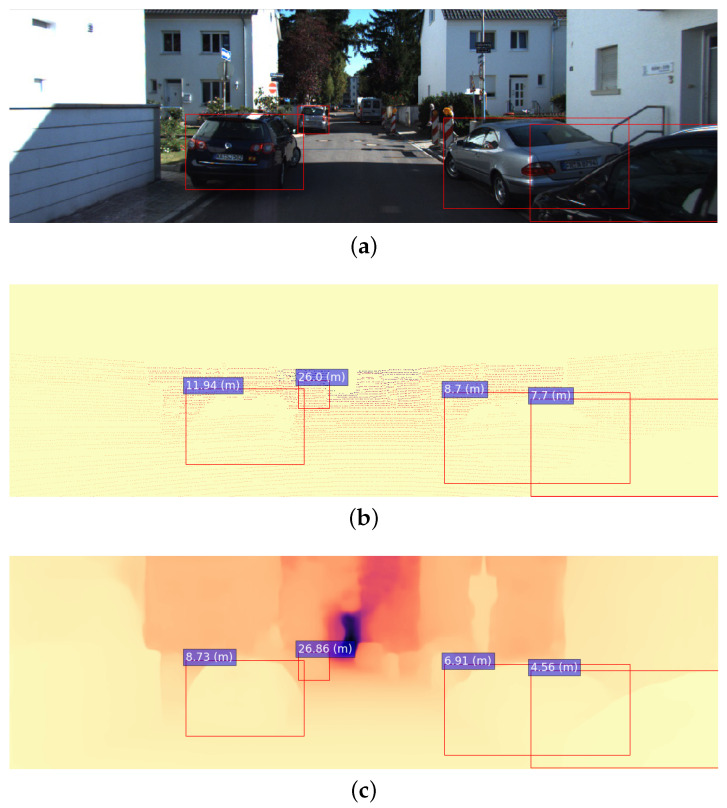
(**a**) A sample image from the dataset with the bounding boxes obtained with YOLOv5; (**b**) its corresponding LIDAR point cloud; (**c**) the depth map generated by Monodepth2. Ground-truth distances and Monodepth2 object distances are shown in blue boxes.

Because the dataset we used is designed mainly for depth map prediction tasks, a sequence of additional actions has to be performed to make it suitable for object-specific distance prediction:

(1) Bounding box generation. This step is performed using the YOLOv5 object detection model. As the model proposed here is intended to be used for autonomous vehicles, only the road agent object classes are considered, namely, “person”, “bicycle”, “car”, “motorcycle”, “bus”, “train”, and “truck” (For simplicity, these classes are referred in the experimental section as C0, C1,…, C6). An image sample with the corresponding bounding boxes can be seen in Figure 1a.
(2) Ground-truth preprocessing. Sparse LIDAR point clouds provided as raw ground truth in the KITTI dataset are converted into distance values via averaging the distance values within object bounding boxes that are applied to the corresponding point clouds. It is important to note that point cloud sparsity affects the precision of the averaging procedure. To address this, we consider only the distance values less then or equal to half the mean distance value during ground truth distance generation. An example of a point cloud with overlapped bounding boxes is shown in Figure 1b.
(3) The same averaging process is applied to the depth maps generated with Monodepth2. We use this data further for comparison during the proposed model’s performance evaluation. Monodepth2 was trained in mono+stereo mode, which enables conversion from disparity to depth maps according to Equation (1).

(1) $$D=\frac{S}{d_{min}+\left(d_{max}-d_{min}\right)d}$$

Here, *D* stands for the resulting metric distance values, while $d_{min}=0.01$ and $d_{max}=10$ are the minimal and maximal disparity values, respectively, S=5.4 is a stereo scaling factor (as described in ref. [2]), and *d* is the initial disparity map generated by the model. An example of a depth map obtained with this procedure is shown in Figure 1c.

After applying this process to the training, evaluation, and testing sets of the Eigensplit, a derivative dataset consisting of 33,328, 1202, and 1658 object samples, respectively, was obtained.

The object class distribution in the training, validation, and testing sets is presented in Table 1. It is evident from the provided numbers that the data are severely imbalanced towards the C2 class (cars), which is to be expected for the real-world image data from the road setting. This imbalance has to be mitigated during the training process by data augmentation and downsampling procedures. We considered the following balancing procedures:

(1) Random oversampling of the minority classes with subsequent augmentation. This procedure was performed in all experiments and consists of randomly copying the members of the minority classes (until the volume of the respective class is enlarged by a factor of 2) and transforming them by randomly applying color jittering, Gaussian blur and adjustment of sharpness (All the transformations were imported from the torchvision module of the PyTorch ecosystem [16]. Augmentation parameters: ColorJitter—brightness = 0.4, contrast = 0.4, saturation = 0.4, hue = 0.2; GaussianBlur—kernel_size = 3, sigma = (0.1, 2); RandomAdjustSharpness—sharpness_factor = 0.7, *p* = 0.5. All other parameters are set to default).
(2) Random downsampling of the majority class. In the experiments that use this technique, the members of the majority class are randomly excluded until the volume of the class is reduced to a fixed number of samples (in all experiments, this number was empirically determined to be 7000).
(3) Near-miss downsampling [17] of the majority class. Unlike random downsampling, this method is deterministic and relies on utilizing a certain distance measure to retain specific members of the majority class. Instead of the widely-used Euclidean distance, average structural similarity (SSIM) [18] is used as the distance measure between the members of the majority and minority classes. This choice is motivated by the better performance of this metric in terms of capturing similarities between image samples. Two versions of the algorithm are considered, where 7000 car samples with the highest average SSIM towards their ten closest (first version, referred to as NM1) or farthest (NM2) neighbors in the minority classes are retained.

### 2.2. Convolutional Object-Specific Depth Regression Model Topology

The proposed model (hereinafter referred to as the CDR Model) consists of four sequential blocks:

(1) **Preprocessing block**: This part of the model performs the image downsizing (in all experiments, the images are downscaled to 640×194 resolution, allowing the use of a smaller feature extractor and consequently increasing computational speed) and object-centered cropping, i.e., extracting a square fraction of the input image with the given side length centered at the midpoint of the bounding box. The side length of the square window is defined as a fraction of the smallest dimension of the input image, rounded up (See Equation (2)):

(2) $$s=\lceil \lambda_{c}\cdot min\left(H,W\right)\rceil$$

Here, $\lambda_{c}\in \left(0;1\right]$ is a hyperparameter regulating the side length (crop factor/cfactor), while *W* and *H* are the input image width and height, respectively.

The main purpose of the cropping operation is to force the network to learn object-specific features, e.g., the objects’ sizes relative to the fixed size of the square window. Additionally, this procedure significantly increases the model’s processing speed.

(2) **Feature extraction block**: To extract the features from the cropped input images, a pretrained convolutional neural network is used, namely, the ConvNeXt architecture [19]. This model has been shown to achieve state-of-the-art results on the ImageNet benchmark and is available in multiple configurations suitable for the inputs of different sizes. We used the small version as a backbone for the CDR model due to the small input dimensions ensured by the cropping performed within the preprocessing block.
(3) **Regression block**: This part of the model consists of seven fully-connected layers with ReLU activation functions. Five of these layers are positioned sequentially and are intended to gradually reduce the feature vector’s dimensionality. The last two layers operate in parallel; one of them yields one or four values (depending on the decoder block type), while the other is used to predict the output distance standard deviation. It is initialized with he Gaussian-distributed random weights with the parameters μ=0,σ=0.0001 (where μ stands for the mean weight value, σ for the standard deviation) and has no bias term. This layer is used when the network is trained with the Kullback–Leibler Divergence Loss Function; the details are discussed below.
(4) **Decoding block**: This block converts the output activations of the neural network into metric distance values. We considered two versions of the decoder: a “dummy” decoder, where a single output neuron of the last fully-connected layer yields the predicted distance value directly, and the optics-based decoder function (“base” decoder) defined in Equation (3), where $f_{x}$ and $f_{y}$ are the camera’s focal parameters, *w* and *h* stand for the bounding box dimensions, and $x_{0},\;x_{1},\;x_{2},\;x_{3}$ are the output activations of the neural network. It is evident from the defined distance function that $x_{0}$ and $x_{1}$ serve as predicted object dimensions in the world reference frame, while $x_{2}$ and $x_{3}$ serve as correction terms to the bounding box dimensions. This decoding scheme provides the network with explicit information about the nature of the task being solved, and therefore increases the training efficiency.

**Figure 2:**
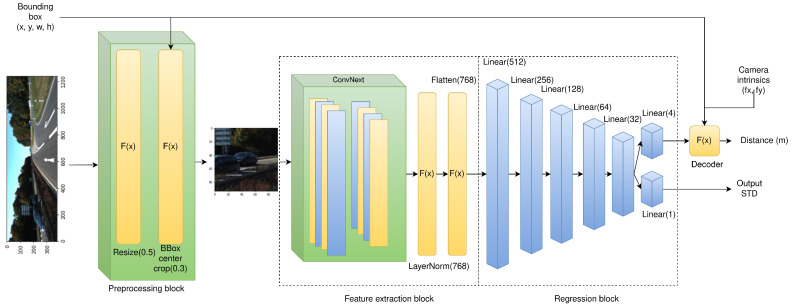
The CDR model topology. The model was trained with a batch size of 512, learning rate of 0.0005 and the Adam optimizer. The version of the network presented in the picture corresponds to the “base” decoder function, using the top output fully-connected layer with four neurons. The model was implemented in PyTorch.

The full topology of the network is presented in Figure 2.

(3) $$z_{pred}=\frac{1}{2}\left(\frac{f_{x}x_{0}}{w(1+x_{2})}+\frac{f_{y}x_{1}}{h(1+x_{3})}\right)$$

### 2.3. Loss Functions

While choosing the appropriate loss function for the task of monocular distance estimation, the following complications must be addressed:

– Classwise imbalance of the dataset. Despite the balancing procedures performed during the data preprocessing stage, further balancing may be needed during the model’s training.
– Randomness introduced by oversampling, downsampling, and imperfect ground-truth data. The errors accumulated due to these factors may strongly affect the standard deviation of the model’s predictions, reducing its overall robustness.

Bearing these complications in mind, we consider two loss functions: Mean Squared Error Loss (denoted as $L_{MSE}$) and Kullback–Leibler Divergence Loss ($L_{KD}$). The former is a well-established choice for regression tasks that possesses the property of penalizing outliers, which may be relevant regarding the specifics of the task. The latter, derived following the process described in ref. [20], is designed to mitigate standard deviation error increase caused by imperfect ground truth data, and may positively affect deviation errors that arise due to the random augmentation procedures. This loss function is implemented according to Equation (4):

(4) $$L_{KD}=H_{1}F_{1}+H_{2}F_{2}$$

Here, $H_{1}$ and $H_{2}=1-H_{1}$ (see Equation (5)) are the Heaviside step functions, which take the difference between predicted and ground-truth distance values normalized by the maximal distance $z_{max}$ as an argument (The maximal distance $z_{max}$ is chosen equal to 80 (m). The same value is used for evaluation of the Monodepth2 model trained in stereo mode; see [2]). The terms perform the switch between L2- and L1-like loss terms depending on the error magnitude. $F_{1}$ is a smooth L1 loss term defined by Equation (6), while $F_{2}$ is the KL divergence term defined in Equation (7).

(5) $$H_{1}=\Theta (|\frac{z-z_{g}}{z_{max}}\left|\right)-1$$

(6) $$F_{1}=e^{-\alpha }\left(\right|\frac{z-z_{g}}{z_{max}}\left|-0.5\right)+0.5\alpha$$

(7) $$F_{2}=e^{-\alpha }{\left(\frac{z-z_{g}}{z_{max}}\right)}^{2}+0.5\alpha$$

Finally, $\alpha =ln(\sigma^{2})$ is the actual value predicted by the standard deviation fully-connected layer to avoid gradient explosion.

To account for the class imbalance, we adopted a weighting procedure based on the effective number of samples [21]. We introduced class weighting according to the definitions in Equations (8)–(10):

(8) $$W=Y_{oh}\cdot w_{c}$$

(9) $$w_{c}={\left(w_{c}^{0}\;\;w_{c}^{1}\;\;w_{c}^{2}\;\;w_{c}^{3}\;\;w_{c}^{4}\;\;w_{c}^{5}\;\;w_{c}^{6}\right)}^{T}$$

(10) $$w_{c}^{i}=\frac{1-\beta }{1-\beta^{n_{i}}}$$

Here, $Y_{oh}$ is a one-hot encoded matrix of class labels, $w_{c}$ is a vector of class weights, and i∈{0,1,2,3,4,5,6}, β∈[0,1]. It is easy to see that β=0.00 corresponds to the class weights equal to 1 (no weighting).

The resulting class-adjusted loss function, i.e., weighted MSE- or KL-Loss, is defined in Equation (11), where the angle brackets denote averaging over a mini-batch.

(11) $$L=\langle W\cdot L_{KD,MSE}\rangle$$

## 3. Results

### 3.1. Hyperparameter Optimization

The initial hyperparameter search for the model was performed manually. During training, 20% of the training part of the Eigensplit was used for validation and early stopping. We used only the validation part of the Eigensplit to estimate the model’s performance to reduce the hyperparameter optimization bias. During the final experiments described below, both the validation and test fractions of the dataset were used to partly account for the insufficient number of samples for certain classes of objects.

In particular, the initial hyperparameter search space included parameters such as:

− *The number of fully connected layers in the regression block*: The dimensionality of the feature vector yielded by the ConvNeXt model (784) has to be decreased to 4 in the output layer. It was observed during hyperparameter optimization that a sequence of five fully connected layers with gradually decreasing output dimensions (from 512 to 32) resulted in lower MAE values compared to other configurations included in the search space;
− *Learning rate*: It was observed that a learning rate lower than 0.0005 resulted in ineffective training, while higher learning rate values increased the output error;
− *Batch size*: A batch size of 512 is a compromise between accuracy and computational complexity. Increasing it further did not show significant accuracy improvements while requiring much more GPU memory;
− *Majority class size after downsampling*: A sample count less than 7000 resulted in underfitting, while a larger sample count led to decreased performance on the minority classes.

### 3.2. Experiments

To explore the variations in the CDR model’s performance depending on its settings, we performed the set of experiments listed below. Each experiment was repeated five times for each model’s setting in order to account for the randomness introduced by the data augmentation procedures and to estimate the standard deviations of the results. The model’s overall performance was measured with two metrics: weighted Mean Average Error (wMAE) and unweighted Mean Average Error (MAE). Weighted MAE was calculated according to Equation (12), where $V_{i}$ is the number of samples of the class *i*, *V* is the total number of samples, and $MAE_{i}$ is the MAE value for the class *i* averaged over five runs. Unweighted MAE was calculated similarly, except with $V_{i}=\frac{1}{7}V$.

(12) $$wMAE=\sum_{i}\frac{V_{i}}{V}MAE_{i},i\in \left\{0,1,2,3,4,5,6\right\}$$

**Figure 3:**
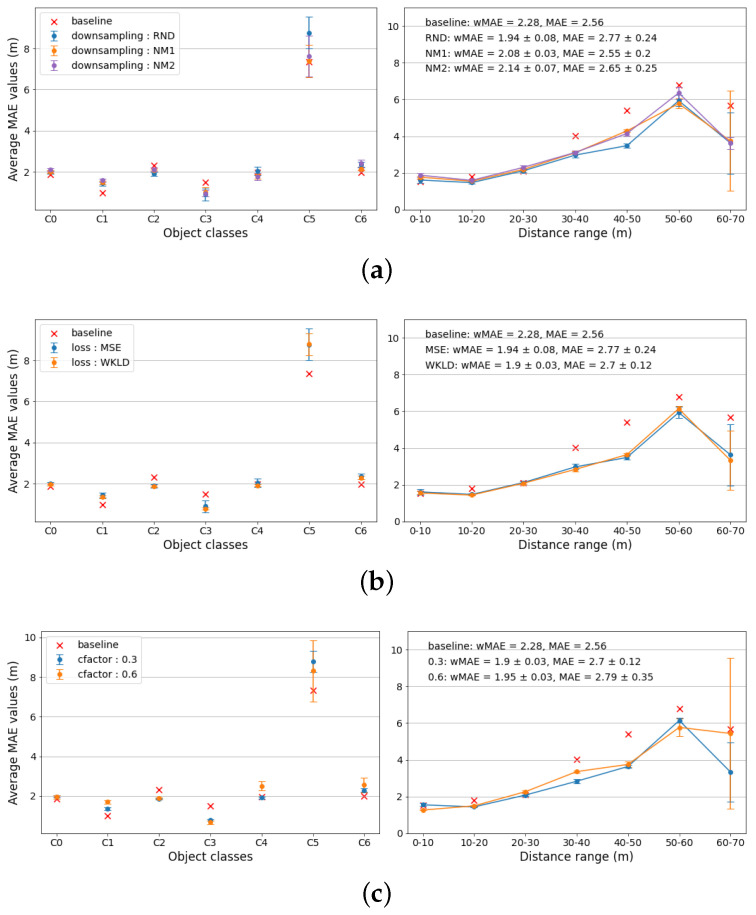
Experimental results for different CDR model settings. The left-hand plots show the MAE values between the model’s predictions and the ground-truth distance values, while the right–hand plots display the MAE metric values distance–wise. Each plot features the baseline (Monodepth2 model) MAE values. Weighted and unweighted average MAE values are shown in the top left corners of the distance plots. Here (**a**) displays the impact of different downsampling strategies on the model’s performance, (**b**)—the impact of the loss function choice, (**c**)—the impact of the $\lambda_{c}$ value.

**Figure 4:**
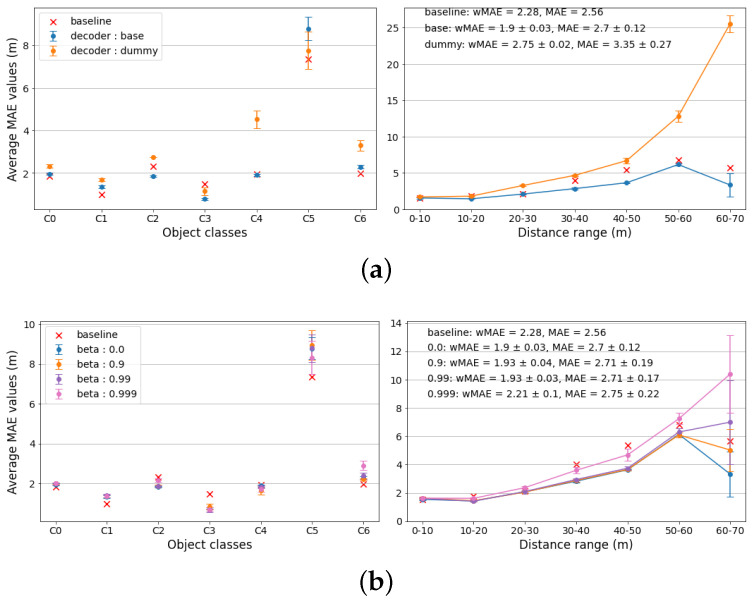
Experimental results for different CDR model settings (continuation). Here (**a**) shows the impact of the decoder function choice on the model’s performance, (**b**)—the impact of the β value.

(1) **Downsampling strategy**. Random downsampling and Near-Miss downsampling are considered. All other model parameters are fixed as follows: β=0.00, $\lambda_{c}=0.3$, MSE Loss, “base” decoder. The results are presented in Figure 3a. As can be seen from the class-wise plot, the random downsampling strategy, while performing significantly worse for class C5 (“train”), performs similarly or better for other classes. The distance-wise plot shows equally similar performance. Considering the metrics, the weighted MAE metric is the lowest for the random downsampling method, albeit exhibiting the highest standard deviation. Considering those facts, in further experiments the random downsampling strategy is used.
(2) **Loss function**. Weighted MSE loss and Weighted Kullback–Leibler Divergence loss (WKLD) are considered. All other model’s parameters are fixed as follows: β=0.00, $\lambda_{c}=0.3$, random downsampling, “base” decoder. While both loss functions perform similarly (see Figure 3b), WKLD loss yields slightly lower weighted MAE value, with significantly lower standard deviation, most likely due to it being designed to minimize deviations in the model’s predictions.
(3) **Crop factor** (cfactor). $\lambda_{c}=0.3$ and $\lambda_{c}=0.6$ are considered. All other model’s parameters are fixed as follows: β=0.00, WKLD loss, random downsampling, “base” decoder. From the plots in Figure 3c, it is evident that a lower crop factor value of 0.3 is optimal for the CDR model.
(4) **Decoder function**. The “Base” decoder (optics-based) and “Dummy” decoder (single output node) are considered. All other model parameters are fixed as follows: β=0.00, WKLD loss, random downsampling, “base” decoder, $\lambda_{c}=0.3$. Thee plots in Figure 4a clearly indicate that the optics-based decoder performs significantly better, validating the hypothesis of simplifying the training process for the model.
(5) **β value**. β∈{0.00,0.90,0.99,0.999} are considered. All other model parameters are fixed as follows: $\lambda_{c}=0.3$, WKLD loss, random downsampling, “base” decoder. The MAE values in Figure 4b suggest that the optimal β values are 0.9 or 0.99, with β=0.99 performing slightly better in terms of standard deviation.

### 3.3. Response Speed Evaluation

Additionally, to compare the CDR model to Monodepth2 in terms of processing speed, we ran both models in inference mode on images from the validation and testing fractions of the Eigen split. For both models, an Intel Core i5-9300H CPU was used. This hardware choice was made to simulate a real-life use case for the model when a GPU is not available. This experiment shows that it takes approximately 0.05 seconds on average for the CDR Model to process a single frame, while the Monodepth2 model takes up to 0.7 seconds to perform the same task.

## 4. Discussion

**Figure 5:**
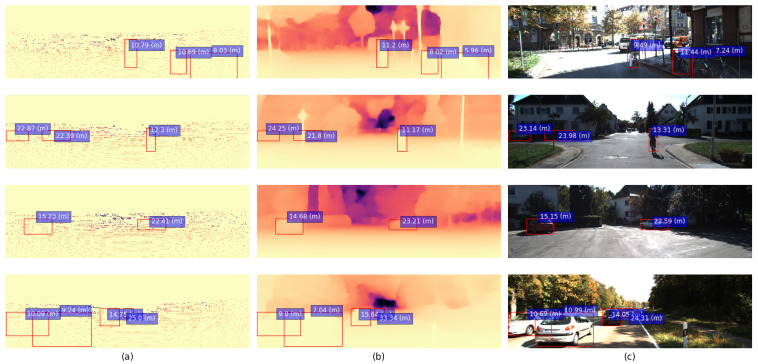
Examples demonstrating the proposed model’s performance on several samples from the validation and testing sets: (**a**) ground-truth LIDAR point clouds; (**b**) corresponding Monodepth2-generated depth maps; (**c**) distances predicted by the CDR model.

Overall, the experiments described above show that the best-performing configuration achieves a weighted MAE metric value of 1.93±0.03 (m) with the following settings: random downsampling of the majority class C2 (“car”), WKLD loss, crop factor of 0.3, “base” decoder. This is 15±1% better than the Monodepth2 baseline, which achieves the wMAE value of 2.28 (m). Examples of the models’ performance can be seen in Figure 5.

However, according to the unweighted MAE metric, the best-performing CDR model configuration performs 6% worse than the baseline (2.71±0.17 (m) against 2.56 (m)). Analyzing the data presented in Figure 3 and Figure 4, it can be concluded that the main source of errors for the CDR model lies in class C5 and at distance ranges above 50 (m). This is to be expected due to the following facts:

– Trains are significantly less common in the road agent setting and can be arguably omitted as a relevant object class. The lack of representation of this class makes errors by an object-specific model higher than for the Monodepth2 model, which is trained in a self-supervised manner based on pose estimations, making it independent of the sufficient presence of a specific object type in the dataset. This is a fundamental limitation of any object-based approach, which can be mitigated by further augmenting the data with artificially generated samples.
– The same argument is valid for objects at higher distances. However, the lack of representation of such objects in the dataset is most likely due to the limitations of the object detection model; therefore, this problem can be addressed by adjusting the confidence threshold for it or by switching to a better-performing alternative.

Apart from directly addressing those limitations, it may be beneficial to incorporate uncertainty estimations of the model’s outputs into its structure. To some extent, this was already achieved: the WKLD loss function operates with variance values predicted by the model, similar to the Bayesian regression loss presented in ref. [22]. According to the authors, a loss function designed in this way reduces both input- (aleatoric) and output-dependent (epistemic) uncertainty. However, the CDR model currently does not leverage Bayesian learning techniques in any other way. Therefore, applying the output variance estimation via variational inference (with Monte Carlo dropout, as described in ref. [23]) can positively affect the model’s robustness.

Having considered the limitations of the CDR model as opposed to self-supervised depth estimation models similar to Monodepth2, it is necessary to consider the advantages that make this study relevant. First, an object-based model is significantly easier to train despite its need for ground-truth measurements; the CDR model was trained using only 1127 images from the KITTI dataset, which is possible due to the large variety of objects in the frames. Second, the CDR Model achieves 20 FPS processing speed while running on a middle-grade CPU, compared to 1.5 FPS for Monodepth2. Finally, an object-based approach arguably has higher generalization potential; training a model similar to CDR on multiple datasets taken with different camera settings is potentially easier due to its explicit use of camera parameters in the decoder and to its focus on object-level features. However, this claim has to be experimentally verified, which will be one of the goals of our further research.

## 5. Conclusions

In this work, we developed an object-specific convolutional neural network that estimates distances towards seven classes of road agents in a real-world road setting.

– In Section 3 it was shown that:
  (1) The best-performing downsampling strategy is random downsampling;
  (2) The weighted Kullback–Leibler loss function (WKLD) results in lower MAE values and significantly reduces the error standard deviation;
  (3) *β* parameter values for an effective number of sample weightings lie within the 0.9–0.99 range, with *β* = 0.99 performing slightly better;
  (4) The optics-based (“base”) decoder function definitively outperforms the “dummy” decoder with a single output node;
  (5) The CDR model in its best-performing configuration yields 15 ± 1% lower average-weighted MAE values than the Monodepth2 model;
  (6) According to the performed inference speed test, the CDR model runs approximately 10 times faster than Monodepth2.
– Among the model’s limitations, the following are apparent from the experiments:
  (1) Despite the balancing procedures, the CDR model performs worse than the self-supervised model on the least represented object classes (i.e., “train”). This is evident from the unweighted MAE metric, which is 6% higher on average for the proposed model. This limitation can potentially be mitigated by using synthetic data;
  (2) The error values increase for the CDR model as the distance grows. This is partly caused by the limitations of the object detector. Therefore, improving this part of the pipeline is likely to improve the results;
  (3) Globally, the error values of the model do not go substantially lower than the 2 (m) mark. This may be a major drawback for the scenarios where an autonomous vehicle is operating in dense traffic.

In essence, we conclude that the proposed convolutional object-specific depth regression model is sufficient for the task of monocular distance estimation and can be used effectively in real-life autonomous driving-related applications. However, taking the above-mentioned limitations into account, we will focus our future research on decreasing the average error further towards the 1 (m) range as well as on improving the results on under-represented object classes.

## Figures and Tables

**Table 1:** Logarithmic class distribution in the training set of the KITTI dataset.

Set	C0	C1	C2	C3	C4	C5	C6
train	3.15	2.77	4.47	2.09	2.37	1.58	3.03
valid	1.62	1.46	3.07	0.48	0.78	0.30	1.67
test	2.06	1.54	3.15	0.70	0.90	1.18	1.86

## Data Availability

The KITTI dataset can be obtained on the official website: https://www.cvlibs.net/datasets/kitti/ accessed on 30 October 2022. This dataset is publicly available and is published under the Creative Commons Attribution-NonCommercial-ShareAlike 3.0 License.

## Abbreviations

The following abbreviations are used in this manuscript:

MDPI	Multidisciplinary Digital Publishing Institute
DOAJ	Directory of open access journals
MAE	Mean Absolute Error
LPG	Local Planar Guidance
DFE	Dense Feature Extractor
CFE	Contextual Feature Extractor
SSIM	Structural Similarity Index Measure
NM1	Near-Miss Algorithm (version 1)
NM2	Near-Miss Algorithm (version 2)
RND	Random (downsampling)
MSE Loss	Mean Squared Error Loss
KL-Loss	Kullback–Leibler Loss
WKLD Loss	Weighted Kullback–Leibler Divergence Loss
cfactor	Crop factor
